# Hsp90: From Cellular to Organismal Proteostasis

**DOI:** 10.3390/cells11162479

**Published:** 2022-08-10

**Authors:** Milán Somogyvári, Saba Khatatneh, Csaba Sőti

**Affiliations:** Department of Molecular Biology, Semmelweis University, H-1085 Budapest, Hungary

**Keywords:** Hsp90, proteostasis, stress, chaperones, development, cancer, neurodegenerative disease, obesity, aging

## Abstract

Assuring a healthy proteome is indispensable for survival and organismal health. Proteome disbalance and the loss of the proteostasis buffer are hallmarks of various diseases. The essential molecular chaperone Hsp90 is a regulator of the heat shock response via HSF1 and a stabilizer of a plethora of signaling proteins. In this review, we summarize the role of Hsp90 in the cellular and organismal regulation of proteome maintenance.

## 1. Cellular Proteostasis

**Figure 1:**
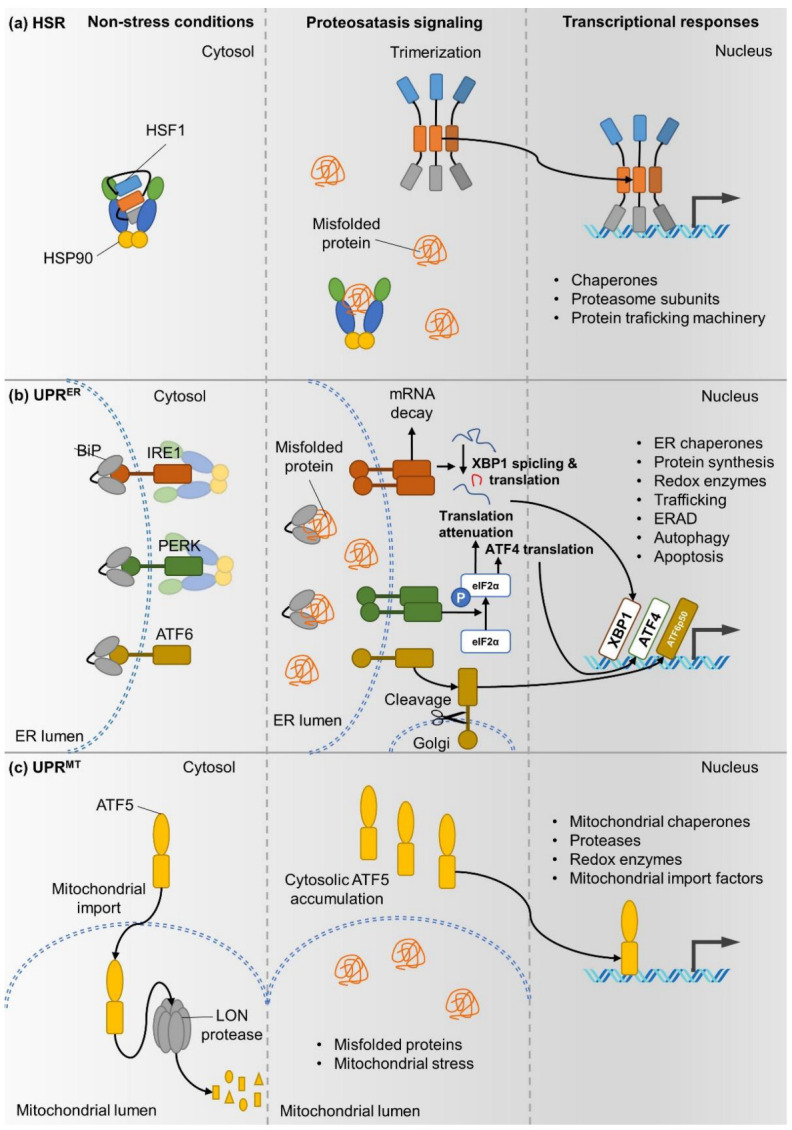
Compartment-specific proteostasis pathways. (**a**) The cytosolic heat shock response (HSR). (**b**) The unfolded protein response of the endoplasmic reticulum (UPR^ER^). (**c**) The mitochondrial unfolded protein response (UPR^MT^). A general theme is the direct detection of and feedback regulation by misfolded proteins.

The mechanism that ensures that the protein pool of the cell is kept in a healthy, functional state is called the proteostasis machinery. Proteostasis is maintained through the cumulative work of multiple cellular processes, starting with protein synthesis, quality control steps that ensure functional conformation and finally, protein degradation through various means. During protein synthesis, “translation factors” are needed for a finely tuned process [1]. As a protein is being produced by the ribosome, it is folded into its three-dimensional conformation. The main components of this machinery are molecular chaperones and their co-chaperones; many of them are heat shock proteins (HSPs). Chaperones themselves do not catalyze any chemical reactions that are necessary for the stability of the functional 3D protein structure, but rather they can relax incorrectly folded parts of the peptide chain, thus making it easier for the polypeptide to find the right energy-minimum state required. Properly folded proteins are targeted with the help of chaperones to places where they can exert their function. Finally, unwanted or erroneous proteins are disposed by protein degradation through the ubiquitin-proteasome system or autophagy, followed by lysosomal proteolysis. The compartmentalization of cellular processes led to the development of compartment-specific proteostasis pathways (Figure 1).

### 1.1. The Cytoplasmic Heat Shock Response (HSR)

The cytosolic protein pool is maintained by the heat shock response (HSR) (Figure 1a). The key regulator of HSR is the highly conserved heat shock transcription factor HSF1. Under normal conditions, HSF1 is kept in an inactive, monomeric form by chaperones Hsp70 and Hsp90. In response to proteotoxic stress, chaperones are recruited to misfolded proteins that accumulate in the cytosol, which releases HSF1. Free HSF1 forms homotrimers, translocates into the nucleus, binds to heat shock elements (HSE), and induces the transcription of various molecular chaperones (heat shock proteins, HSPs), trafficking and proteolytic genes, which in turn restore cytosolic protein homeostasis, either by refolding or degrading misfolded proteins [2]. HSF1 is also regulated by multiple post-translational modifications (PTMs). For example, the SIRT1 deacetylase is able to prolong promoter-binding of HSF1 by removing an inhibitory acetyl group [3]. This points to a necessity for fine-tuned activation of the HSR through HSF1 in order to exert a proper response to any proteotoxic harm that might appear in the cytosol.

### 1.2. The Unfolded Protein Response of the Endoplasmic Reticulum (UPR^ER^)

The endoplasmic reticulum relies on its own protein maintenance program, the so-called unfolded protein response (UPR^ER^) (Figure 1b). Protein misfolding in the ER is sensed through three conserved pathways, starting from the transmembrane sensor proteins IRE1α, PERK and ATF6α. These proteins sense misfold proteins in the ER lumen either directly or via forming a complex with the ER Hsp70 paralog BiP. They are activated in response to proteotoxicity. The PERK kinase phosphorylates eukaryotic translation initiation factor 2 subunit-α (eIF2α), and thus shifts translation from general protein synthesis to ATF4 translation. PERK was found to be among those proteins that require Hsp90 for their stabilization [4]. The IRE1α RNase, once activated, excises a 26bp intron from the mRNA of X-box-binding protein 1 (XBP1) transcription factor, thereby leading to the production of its active form. Besides PERK, IRE1 was also shown to be associated with Hsp90 by its cytoplasmic domain and is considered to be a Hsp90 client [4]. Hence, both UPR pathways depend on Hsp90 for their functionality. ATF6 itself is a “proto-form” of a transcriptional factor. Upon ER stress, it translocates to the Golgi-apparatus and is cleaved and activated by a site-1 protease. ATF4, XBP1 and ATF6 induce the expression of gene ER protein folding, proteasome, autophagy and apoptosis to combat with ER stress.

### 1.3. Mitochondrial UPR (UPR^MT^)

The extensive proteome of the mitochondria is maintained by a finely regulated process, the mitochondrial unfolded protein response (UPR^MT^). A key component of UPR^MT^ is the bZIP transcription factor ATF5. Under normal conditions, ATF5 is degraded after being imported to the mitochondrion. Mitochondrial stress leads to inhibition of the ATF5 import; thus, the transcription factor can accumulate in the cytosol and enter the nucleus, where it induces multiple genes. Among these are chaperones and proteases, reactive oxygen species (ROS) detoxification enzymes and factors that increase mitochondrial import, leading to restoration of mitochondrial proteostasis and functionality.

## 2. The Hsp90 Chaperone

### 2.1. Hsp90 Isoforms and Structure

The 90-kDa heat shock protein Hsp90 is an essential evolutionary conserved molecular chaperone. It exists in all living organisms, except archaea. Under normal conditions, it is one of the most abundant members of HSPs and represents 1–2% of all cellular proteins [5]. Besides the cytosolic Hsp90, other Hsp90 paralogs are located in the endoplasmic reticulum (Gp96 or Grp94), and in mitochondria (TRAP), as reviewed elsewhere [6,7]. Hsp90 already appears in bacteria as HtpG (high temperature protein G) [8]. HtpG is not essential, rather it is required for growth at high temperatures [8]. Human Hsp90 shares 50% sequence similarity with the *Escherichia coli* HtpG [8]. Simple metazoans, such as *Caenorhabditis elegans* (HSP-90, previously DAF-21) and *Drosophila melanogaster* (Hps83), have only one cytosolic Hsp90, whereas the yeast *S. cerevisiae* [9] has two, includin the constitutive Hsc82 and the heat-inducible Hsp82 [10,11,12]. Despite their 97% amino acid identity [10], there are differences in client preference as well as in their sensitivity to Hsp90 inhibitors [13].

Mammals also possess two cytoplasmic HSP90 isoforms, the stress-inducible isoform HSP90α (HSP90AA1 and HSP90AA2), and the constitutive isoform HSP90β. Although Hsp90 isoforms are 85% homologous in sequence [9] with functional similarity, they have distinctive functions, such as differential binding to client proteins and their role in cell differentiation and development [14]. Dimerization is vital for the Hsp90 ATPase activity [15]. Hsp90α shows more tendency for dimerization than Hsp90β [14]. Nevertheless, the phosphorylated dimerized form of Hsp90 contains 2–3 covalently bound phosphate molecules per monomer [6] and is found to be in an elongated form [16]. Hsp90 possesses ATPase activity, but is also able to hydrolyze GTP, especially in the presence of calcium [17]. Heat shock leads to an increase in its hydrophobicity [18].

**Figure 2:**
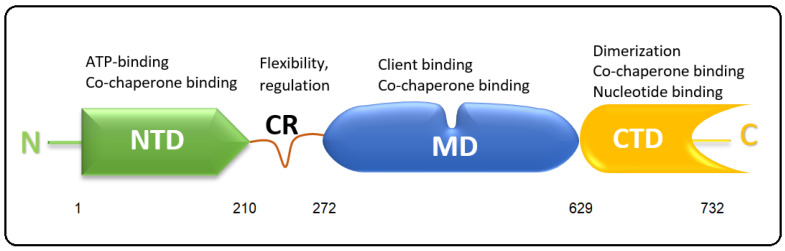
A schematic representation of the Hsp90 domain structure. NTD, N-terminal domain; CR, charged linker region; MD, middle domain; CTD, C-terminal domain. Numbers indicate the amino acids at the domain boundaries.

The molecular structure of Hsp90 consists of the following three main conserved domains: the N-terminal nucleotide-binding domain (NTD), the client-binding middle domain (MD), and the C-terminal dimerization domain (CTD) [5,19] (Figure 2). In addition to the three main domains, eukaryotic Hsp90 has a highly charged linker region (CR) [20] that plays a crucial role in the chaperone function, interaction and flexibility [20,21].

Each Hsp90 domain executes specific functions. The NTD forms a highly conserved GHKL-type nucleotide binding domain [22], which is also found in bacterial topoisomerases. The domain is rich in beta strands and it shows a tenfold higher affinity to ADP than to ATP. Hsp90 exhibits a slow but activatable ATPase activity that is necessary for the chaperone cycle and binding to the client proteins. It is the target of Hsp90 inhibitors, such as geldanamycin, radicicol and various novel compounds, which compete with the ATP for the ATP binding site. The MD is involved in binding to client proteins and various co-chaperones, such as Aha1, Cdc37 and CHIP. These events on the MD also regulate the ATPase activity of Hsp90, partly by the dynamic transitions of the CR. The MD also has a domain for calmodulin binding [23]. The CTD domain contains a site for Hsp90 homodimerization [24] and binds nucleotides [25,26,27] and is targeted by C-terminal Hsp90 inhibitors. It also carries special motifs depending on the HSP9 isoforms and its cellular location [14]. Cytoplasmic Hsp90 carries the MEEVD motif in both isoforms at the CTD, which interacts with tetratricopeptide repeats (TPR) containing co-chaperones [28], such as Hop and the protein phosphatase PP5 [9]. The CTD also serves as an allosteric regulator of the NTD ATPase activity.

### 2.2. Hsp90 Function

Hsp90 assists with the general folding and stabilization of proteins that are under stress, thus maintaining proteostasis and cell survival [29]. In human cells, it was estimated that 60% of the kinases are dependent on Hsp90 [30]. In addition, 30% of E3 ubiquitin ligases and 7% of the transcription factors are found to interact with Hsp90, such as p53 transcription factor [30]. A comprehensive, up-to-date list of clients can be found at www.picard.ch/downloads/Hsp90interactors.pdf (accessed on 19 May 2022). Most of these clients are important for various biological signaling pathways. After creating a functional map [31] for an overview of the function of Hsp90 by analyzing its interactome, they identified development, signaling, and cell cycle as the major biological functions of Hsp90 as they formed a cluster in a distinct region. Surprisingly, stress response and protein folding functions were represented in a smaller cluster. In addition, other Hsp90-associated functions include nucleocytoplasmic transport, DNA repair, and immune response. However, Hsp90 function might go beyond that. It is one of the most connected proteins in the cell; in yeast, it interacts with cca. 20% of the proteome [32,33]. In addition, Hsp90 inhibition induces the leakage of the cellular protein content from the cell [34]. These facts suggest that Hsp90 is a pivotal factor in topological proteome stability and cellular integrity.

No common binding site/motif was identified on Hsp90 clients, as in the case of the common extended and accessible hydrophobic sequence that is found in Hsp70 substrates [12,35]. On the other hand, many factors are available that explain the dependency of the clients on the Hsp90, including a large multidomain structure, folding cooperativity, overall instability and thermodynamic instability, as well as reduced compactness [12].

Hsp90 functions are client-dependent, as it performs different roles with different client proteins. Hsp90 is important for maturation and also for maintaining a specific activatable state, as is the case in some kinases [36,37,38]. It maintains stability and facilitates ATP binding to the kinase [39]. Hsp90 also helps clients in the assembly of protein complexes, such as the kinetochore. Steroid hormone receptors (SHR) are also examples where Hsp90 action favors ligand binding [40,41,42].

### 2.3. The Hsp90 Chaperone Cycle and Its Regulation

The chaperone cycle of Hsp90 helps fold and stabilize its client proteins [7]. The activity, selectivity, and functionality of Hsp90 are regulated by co-chaperones, as they intervene at different stages of the Hsp90 activation cycle [43]. The Hsp90 chaperone cycle is intimately connected to the binding and hydrolysis of ATP. After binding the high-energy nucleotide to the binding cleft in the NTD, Hsp90 undergoes a series of conformational changes.

For Hsp90 to properly fold a misfolded or newly synthesized protein, the client first forms a complex with Hsp70/Hsp40/ADP, which helps to order and protect aggregation [44]. Additional co-factors, such as Bcl2-associated athanogene homologs or Hip, can further stabilize the complex and later facilitate the ADP/ATP exchange [45]. Hsp90 binds to the client protein in the complex. The p50/cdc37 kinase-targeting cochaperone facilitates the binding of kinases, whereas p23 facilitates nuclear receptor binding to Hsp90. A homodimer forms, while Hsp70, Hip and Hop are released [46]. ATP binds to the NTD of Hsp90, changing its conformation from the open to the closed state [47]. Other cochaperones, such as Aha1, will bind to the MD, which induce ATP hydrolysis and P_i_ release that facilitates the folding and the release of the cochaperones and immunophilins [48] and the return of Hsp90 to its original “open state” [49]. Some clients bind to Hsp90 with a moderate affinity [42,50,51], while for other clients, the affinity is affected by Hsp90 conformation, as in the case of glucocorticoid receptors (GR). Some clients of Hsp90, such as p53, showed a dynamic interaction and contacts with the MD and CTD [52,53]. N-terminal Hsp90 inhibitors (such as geldanamycin, radicicol and their derivatives) bind to the ATP-binding site in NTD and inhibit the ATPase activity [54,55].

Hsp90 exhibits various post-translational modifications, such as phosphorylation, acetylation, methylation, ubiquitination, and SUMOylation [56,57]. The role of these modifications is not fully understood. Phosphorylation of Hsp90 occurs predominantly on serines, but also on threonine and tyrosine residues [58]. Phosphorylation slows down the conformational cycle of the Hsp90 and affects the maturation of the clients and the interaction with the co-chaperones [59]. Interestingly, hyperphosphorylation leads to impairment of the client maturation process [60]. Hyperacetylation of Hsp90 inhibits the binding of the p23 co-chaperone [61,62]. A negative feedback mechanism through PTM of Hsp90 was suggested on the protein endothelial nitic oxide synthase (eNOS). Here, S-nitrosylation of Hsp90 occurs at a cysteine in the CTD [63], which in turn inhibits the ATPase activity and impairs eNOS activation by Hsp90 [64]. In order for Aha1 to bind to the MD of Hsp90α, the highly conserved region Tyr313 of MD needs to be phosphorylated first, so that Aha1 can be recruited to the C-domain and as a result, it can stimulate the ATPase activity of Hsp90 [65]. Thus, it appears that Hsp90 function and interactions are tightly controlled by numerous PTMs.

## 3. Hsp90 in Intracellular Proteostasis

Hsp90 has been largely recognized as an intracellular molecular chaperone responsible for stabilizing a growing number of client proteins, and regulating HSF1, the master regulator of proteostasis (Table 1). HSF1, while being responsible for the induction of Hsp90 expression itself, is also regulated by other stress-responsive pathways, such as the FOXO transcription factor, or the SIRT1 protein deacetylase. These two, with a reputation as potent stress-response factors on their own, were also suggested to be regulated by Hsp90. In the nematode *C. elegans*, Hsp90 is required for the translocation of the FOXO ortholog DAF-16 isoform A into the nucleus in response to heat stress and dampened IIS, and for its function in the determination of normal and extended lifespan [66]. As DAF-16 is also an important regulator of proteostasis, further studies might reveal how Hsp90 affects its function in proteotoxic conditions. One connection might be that in a volatile toxicity model, both DAF-16 and Hsp90 were required for physiological and behavioral tolerance against benzaldehyde, a protein adduct forming oxidative agent [67]. The SIRT1 deacetylase binds to Hsp90, and proteasomal degradation of SIRT1 occurs in response to Hsp90 inhibition or knockdown in both nematodes and mammalian cells [68]. Thus, SIRT1 (and its *C. elegans* ortholog SIR-2.1) are also clients of Hsp90.

Besides PERK, three other kinases are responsible for eIF2α-phosphorylation, including GCN2, PKR and HRI. These four kinases form the so-called integrated stress response (ISR). All four of these proteins depend on Hsp90 for their stability [4,69,70,71], highlighting the importance of Hsp90 in ensuring the proper responsiveness of the cell to various stresses. Hsp90 is not only responsible for the attenuation of the effects of proteotoxic stress, but was also shown to be an essential component of the secretory pathway and cellular transport, indicating an important role in cellular protein traffic [72]. These activities associated with Hsp90 are able to dynamically respond to various stimuli. The capacity of Hsp90 to stabilize unstable proteins is largely dependent on the state of the proteome; proteostasis stresses can lead to overload of the Hsp90 chaperone machinery, resulting in HSF1 dissociation and activation, hence upregulating Hsp90 levels. However, clients might also dissociate, lose their stability and function, and become degraded eventually.

The cellular molecular chaperone pool, including molecular chaperones, co-chaperones and other co-factors, was first dubbed the “chaperome” in 2006 [73], meaning that the state of the folding machinery in the cell that can change in response to proteotoxic and proteostatic effects. Later, this concept was greatly expanded upon by the works of Gabriela Chiosis and colleagues. Their approach focused on multimeric stable chaperome complexes that are able to exert distinct functions under stressful conditions, becoming more tightly and strongly interconnected with the level of stress and differing from the canonic folding cycles associated with single chaperone molecules and their targets [74]. The extreme dependence of tumor cells on a hyperconnected chaperome network points to an opportunity for novel approaches to cancer treatment. Whereas a healthy chaperome is able to dynamically respond to perturbations of most, if not any, of its members, a hyperconnected chaperome present in chronically stressed and tumor cells is more rigid, unable to cope with more challenges and might even collapse if a key chaperome network unit is compromised. Hsp90 is a key member of various chaperomes with different protein compositions and distinct functions. These different chaperomes may not even differ in their composition and the strength of their connectivity, but this might also affect the kinetics, and thus the efficacy, of small molecule modulators [75]. In the study it is argued that the efficacy of the inhibitor molecule is largely influenced by kinetic factors and not by its thermodynamic binding preference. Furthermore, the hyper-connectedness or rigidity of the chaperome might have profound consequences on the sensitivity of cancer cells to Hsp90 inhibition; a less interconnected proteome means that disturbing Hsp90 function might affect a subset of the regulated network, rendering Hsp90 non-essential, while a hyper-connected “epichaperome” has increased sensitivity towards the inhibition of Hsp90. Sensitizing tumors to Hsp90 inhibition before treatment might be an avenue worth pursuing in the future.

## 4. Hsp90 in the Extracellular Space and Proteostasis

Hsp90 is not only essential inside the cells, but also has important extracellular functions (Table 1). Of the two mammalian isoforms of Hsp90, Hsp90α and Hsp90β, only the former is induced by heat shock and mostly, it is only found in the extracellular space [95]. Various environmental insults trigger cells to secrete Hsp90. Reactive oxygen species [96], heat [97,98], γ-irradiation [99], hypoxia [100,101], injury-released growth factors [102], serum starvation [103] and virus infection [104] are among them. It appears that extracellular Hsp90 (eHsp90) is one of the messages stressed or that injured cells emit. Tumor cells, however, tend to secrete Hsp90 constitutively, due to them being constantly under stress from the overdriven growth pathways, as well as in response to serum starvation [103], possibly through TGF-β signaling [105], thus promoting tumor cell migration and invasiveness [106]. eHsp90 can travel free from, or within, extracellular vesicles (EVs) [107]. Since Hsp90 does not have a secretory signal sequence, it probably utilizes a non-canonical pathway in order to reach the extracellular space [108]. Multiple modes were proposed to explain the secretion of Hsp90 from the cell, including removal of its proposed cytoplasmic retention sequence [109], using secretory lysosomes [110], or EVs together with other chaperones and co-chaperones [107,111]. The participation and interaction of these pathways in eHsp90 release remains to be investigated. eHsp90 has clients outside the cell in the extracellular matrix (ECM). Some of its extracellular clients are the matrix metalloproteinase 1 (MMP1) [91], MMP2 [92], MMP3 [93] and MMP9 [94], responsible for promoting tumor cell invasion and metastasis formation through degrading ECM proteins, such as the various types of collagen, fibronectin and laminin. Recently, it was reported that the tissue inhibitor of metalloproteinases-2 (TIMP2), which acts as a stress-inducible co-chaperone, inhibits MMP2 by loading it to eHsp90, while inhibiting the ATPase activity of the chaperone, thus keeping the protease in an inactive form. Another co-chaperone, the previously mentioned Aha1, is able to displace TIMP2 and ultimately reactivate MMP2 [87].

**Figure 3:**
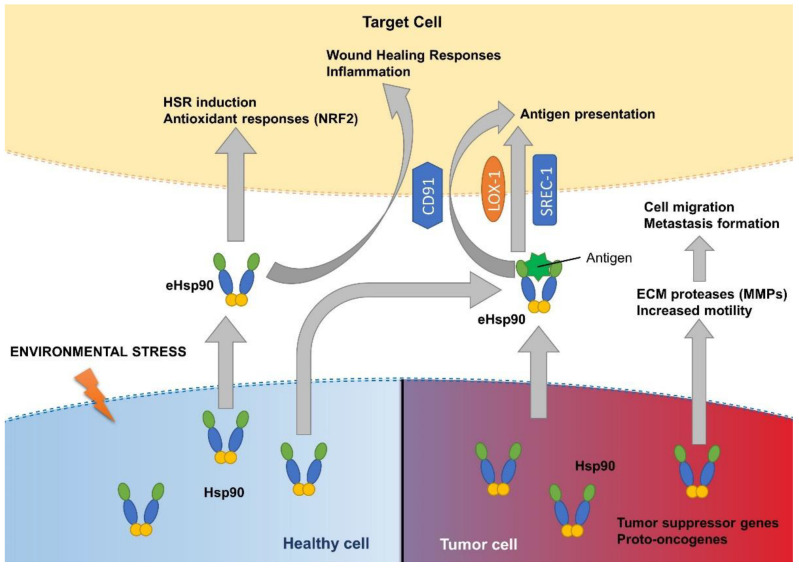
Extracellular functions of Hsp90. Hsp90 is secreted in response to various stresses from normal or from tumor cells. Outside the cell, eHsp90 exhibits various functions, including wound-healing and inflammation, antigen presentation, antioxidant signaling, or restructuring of the ECM, promoting cell migration and metastasis formation.

eHsp90 needs to be recognized by the cells it aims to affect. To date, multiple scavenger receptors (such as LOX-1, SREC-I and LDL binding receptor CD91) were shown to bind to a wide range of chaperones [84,112], and their cell type-specific expression ensures differentiated responses. Extracellular Hsp90 was shown to be involved in immune [85] and inflammatory responses [113], as well as in the antioxidant stress response [114] (Figure 3). For instance, eHsp90 can capture intracellular antigens and present them to an antigen presenting cell (APC) through receptors SREC-I and LOX-1. eHsp90 can also activate wound healing responses through binding to CD91 [115] in human dermal fibroblasts and pig skin as a model for wound healing. It was also observed that when Hsp90 enters a microglial cell, it is able to switch on an antioxidant response pathway through the activation of Nrf2, deter inflammation, trigger autophagy of phagocytosed materials, and thus protect adjacent neuronal cells’ proteome from the potential harm caused by an oxidative burst [114].

It was reported that vesicular secretion of HSPs is able to improve proteostasis in a cell non-autonomous manner in worms [116], flies [107] and fibroblast cell lines [117]. Addition of Hsp40/Hsp70-containing exosomes efficiently suppressed inclusion body formation in polyglutamine-expansion expressing cells; therefore, exosome-mediated transmission of chaperones is able to cell non-autonomously improve the protein-folding environment [107]. This suggests that extracellular chaperones, including eHsp90, play a similar role, i.e., they improve proteostasis by supporting the proteins/cells in need at the level of the tissue. Similarly to the above described intracellular chaperome concept, it was suggested that eHsp90 might form various chaperone complexes outside the cell with specific functions [118]. The nature of these complexes and their function remains poorly understood as of yet, but investigating them promises new diagnostics tools and might even provide potential novel targets for drug interventions in pathologies such as cancer.

## 5. Hsp90 Is a Signal and Executor in Organismal Proteostasis

In *C. elegans,* two thermosensory AFD neurons are responsible for temperature-dependent behavior via the AFD-specific receptor-type guanylyl cyclase *gcy-8* [119]. Lack of this receptor dampened the HSF1-dependent induction of the HSR in non-neural tissues in a temperature-specific manner, leading to a failure to restore proteostasis and weakened thermotolerance (i.e., survival at lethal heat stress) [120], showing neuronal control of cellular proteostasis. HSF1, on the other hand, is able to transmit information about temperature changes in the non-neuronal cells to the heat-sensing AFD neurons through estrogen signaling, using nuclear hormone receptor 69 (NHR-69) [121]. A similar feedback-loop was identified in *Drosophila*; FOXO activity in muscle cells was able to limit insulin secretion from neurosecretory cells, which then delayed the age-related build-up of protein aggregates in distant tissues [122]. The *C. elegans* ortholog of FOXO also drives the stress resistant diapause of worms in the dauer larval stage, where the proteostatic buffer capacity is greatly enhanced. A neural integrator of dauer diapause in response to temperature, food availability and population crowding is the TGF-β receptor [123], which was shown to depend on Hsp90 [124]. The UPR in the endoplasmic reticulum and in mitochondria were also shown to be regulated cell non-autonomously in mammalian cells. ER or mitochondrial stress in neurons led to UPR induction in not affected, non-neuronal cells [125,126]. Another cell non-autonomous signal that affects organismal proteostasis comes from germline stem cells (GSCs) [127]. It was found that GSC signaling leads to the age-dependent decline in proteostasis through multiple different signaling pathways, such as *hsf-1* and *daf-16*, *daf-12*, *daf-9*, *daf-36*, *nhr-80* and *pha-4*, all involved with protein maintenance in the soma. The above examples illustrate a coordination of proteostasis mechanisms in neighboring tissue cells or throughout the whole organism.

Is it possible that an Hsp90 imbalance in one tissue might somehow relay signals to other tissues? In roundworms, the expression of large chaperones, such as Hsp70 and Hsp90, are not only induced through neuronal signals, but also by changes in Hsp90 capacity in muscle or gut tissue. For instance, muscle-specific reduction in Hsp90 availability by the expression of misfolded proteins upregulates Hsp90 in other tissues, which confers protection from the debilitating effect of the misfolded proteins. Interestingly, the expression of Hsp90 in other tissues also confers protection. However, since Hsp90 exerts an inhibitory effect on HSF1, general induction might have detrimental effects under heat shock. In contrast, when Hsp90 is knocked down in certain tissues, a compensatory HSF-1-activation mechanism leads to elevated Hsp70 levels in distant tissues in a FOXA-dependent manner [128]. Thus, local Hsp90 capacity is sensed via “transcellular chaperone signaling”. Such organismal consequences of local HSR induction were also observed in mammals through neuroendocrine regulation; stressed animals have higher levels of cortisol, which is secreted by the pituitary gland, leading to increased trimerized HSF1 with higher DNA binding activity, resulting in increased levels of the Hsp70 in the adrenal glands [129]. However, sufficient Hsp90 capacity is needed to ensure the activation of the glucocorticoid receptor in the target cells. PQM-1 has recently joined the other well-known proteostasis regulators. It was first observed to be involved with oxidative stress response, since exposure to paraquat lead to its induction [130]. Its importance in thermotolerance and proteostasis during ageing has only been demonstrated recently [116,131]. The transcellular chaperone signaling also requires PQM-1; tissue-specific overexpression of Hsp90 triggers PQM-1 activation in neurons and the intestine, leading to cell non-autonomous Hsp90 upregulation through direct or indirect ways [116].

## 6. Hsp90 Is a Capacitor of the Evolution of the Proteome

Landmark studies of Susan Lindquist and colleagues showed that Hsp90 acts as an evolutionary scaffold, allowing the development of metazoan signaling and morphogenesis. In 1998, Rutherford and Lindquist, working with *Drosophila* Hsp90 (Hsp83), unraveled the phenomenon where Hsp90 was able to mask the phenotypic effects of mutations by stabilizing the altered polypeptide chains [132]. The idea behind this is that a high Hsp90 buffer capacity (providing a large chaperone pool) allows for many destabilizing variations in the organism, or in the population, without their effect becoming apparent. Various stresses, however, lead to proteostasis imbalance and the accumulation of misfolded proteins overload Hsp90 capacity. This, in turn, releases the variant proteins from Hsp90, revealing the effects of mutations. This mechanism was suggested as one potential molecular basis of sudden evolutionary changes [133]. The concept of canalization accounts for a trait becoming stable in the face of genetic or environmental perturbation. Canalized traits are the cause behind the remarkable phenotypic robustness of wild-type organisms. This can be advantageous in constant environments, but in the case of an environmental shift, extreme canalization can also be a disadvantage, since it leads to “rigid” responses to changes [134]. By being able to cover up genetic diversity in a context-dependent manner, Hsp90 is able to open up a path to de-canalization, thus leading to more phenotypic diversity in response to environmental challenges.

**Figure 4:**
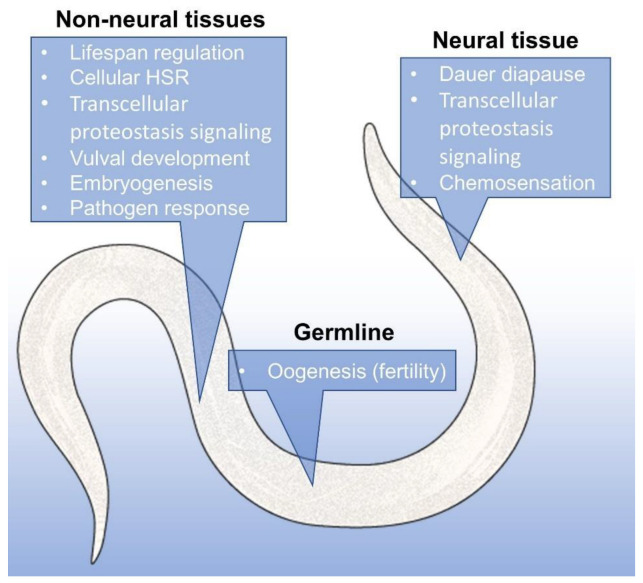
Hsp90-dependent organismal processes that involve proteostasis in *C. elegans*.

**Figure 5:**
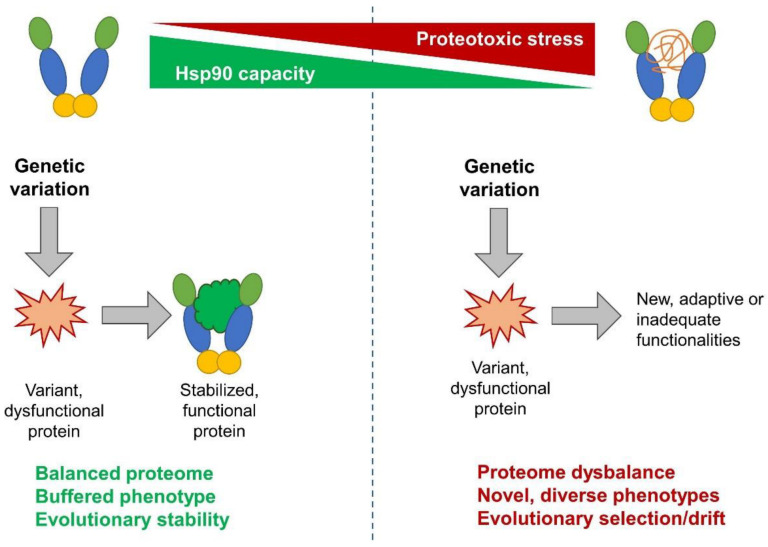
Hsp90 ensures proteome stability and acts as an evolutionary capacitor. Adequate Hsp90 capacity masks the effects of genetic variation by stabilizing mutant proteins and ensuring their functionality. Stressful conditions reduce Hsp90 capacity to inadequate levels, which releases cryptic variations, which may result in sudden changes in protein networks and in the phenotype. Non-adaptive changes are purged, whereas adaptive ones are selected by natural selection. Thus, Hsp90 is a stress-responsive regulator of evolutionary adaptation of the proteome.

The penetrance of this phenotypic diversity largely depends on the robustness or capacity of the HSR, mostly Hsp90, as shown in a study using *C. elegans* [135]. The involvement of Hsp90 in the various life processes of *C. elegans* is shown in Figure 4. This dependency was proposed to be a kind of trade-off between stress resistance and higher reproductive fitness [136], where individuals with higher stress resistance may mask more mutations, leading to more homogenous progeny and a stable population, while lower stress resistance unlocks the potential for better survival through the previously unobserved variations in the next generation. If these mutants, in turn, are able to better respond to the stresses, a continued selection pressure can stabilize the new trait through the process of genetic assimilation [137] (Figure 5). The observed variations tend to be related to clients of Hsp90; they are either clients themselves, interactors of clients, or even regulatory regions that are bound by clients [138]. Disturbances in Hsp90 function can contribute to evolutionary change in other means as well; since many clients of Hsp90 are involved in ensuring the stability of the genome, it directly follows that a reduction in Hsp90 capacity may lead to genomic instability, resulting in an elevated frequency of mutations of microsatellites [139] and increased transposon mobility [140]. There is evidence that supports the idea that Hsp90 might also affect the chromatin and gene expression [141,142], but its effects seem to be complex and locus-specific, due to the diversity of its client proteins. Hsp90 capacity-loss was also brought into association with the emergence of complex birth defects [143], and even cancer [144], where it was proposed that among the mutations masked by Hsp90 are many that might be oncogenic in nature; thus, their phenotypic effect on malignant transformation can be induced by stress.

Since Hsp90 keeps these oncogenic mutant proteins in a functional form, protecting it from degradation, this gives an additional angle to the development of Hsp90 inhibitors in the treatment of cancer.

## 7. Hsp90 as a Target in Diseases of Proteostasis

Obesity is becoming an increasing health risk in the developed world. A key regulator of adipogenesis is the peroxisome proliferator-activated receptor-γ (PPARγ), which was also found to be a client of Hsp90 [145]. Transient heat shock, the archetype of proteotoxic stress, reversibly destabilized PPARγ and halted adipogenesis, showing that proteostasis regulates tissue differentiation and function via the Hsp90-client connection. These results were later supported by a study carried out using a mouse model of nonalcoholic fatty liver disease (NAFLD), where Hsp90 overexpression resulted in increased PPARγ signaling, while inhibition of Hsp90 reduced PPARγ protein levels and signaling [146]. In a recent study using ovariectomy (Ovx) rats, it was observed that the obesity induced by ovariectomy was reversed by treatment with the Hsp90 inhibitor 17-allylamino-17-demethoxygeldanamycin (17-AAG). The treatment also resulted in an increase in the expression of Hsp70 and the phosphorylation of Akt [147]. The above findings indicate that proteostasis has a profound effect on the organism through Hsp90 and its cellular client PPARγ, since the adipose tissue, besides being an energy storage organ, has endocrine and immune functions as well.

Tumor cells, due to their increased proliferation, the intensive utilization of growth signal pathways, along with the various proteostatic and metabolic stresses, are more reliant on Hsp90 than normal cells. More than 400 Hsp90 clients are involved in cancer signalization, while extracellular Hsp90 is also associated with tumor formation through MMPs, as shown below. The presence of dysfunctional proteins triggers the canonical HSR in order to keep the cell’s proteome in a functional state. This fact alone is enough to underline the relevancy of Hsp90 inhibition research in the therapy of neoplastic diseases. However, besides this canonical role, many specific clients of Hsp90 were found to be involved in the pathogenesis of cancer. Among other HSPs, Hsp90 is also considered to be a target in the therapy of osteosarcoma (OS) [148]. HSPs are overexpressed in multiple cancer types, such as breast, prostate, colorectal, lung cancers and OS [149]. Hsp90 itself was shown to promote tumor growth [150], as well as metastasis formation with the involvement of the co-chaperone Aha1, which upregulates another Hsp90 client, isocitrate dehydrogenase 1 [150,151]. Hsp27, Hsp60 and Hsp70 were also shown to have a role in the regulation of these processes, mainly the promotion of OS tumor cell proliferation. The involvement of multiple heat shock proteins in the progression of OS also raises the possibility of targeting HSF1 itself in cancer therapy. Therefore, Hsp90 inhibition is considered a valuable single hit-multi target approach in cancer therapy. Multiple inhibitory molecules have undergone a rigorous evaluation process and some reached the phase of clinical trials. Among these are 17-AAG, an analog of geldanamycin [152], resulting in pro-differentiation and anti-chemoresistance effects; PF4942847 [150], an anti-proliferation and pro-apoptotic agent; Ganetespib [153], leading to tumor regression; and finally SNX-2112 [154], also with anti-proliferation and pro-apoptotic effects. Some of the inhibitory compounds are N-terminal ATP-competitive inhibitors, such as geldanamycin and its analogs (17-AAG, 117-DMAG, ganetespib etc.), radicicol [155,156], PU-H71 [157], while others are allosteric C-terminal inhibitors (such as novobiocin and chlorobiocin) [25,44], or even allosteric activators of Hsp90 (for example dihydropyridines) [158,159,160].

The very cause of neurodegenerative diseases, such as Alzheimer’s disease, Parkinson’s disease (PD), and Huntington’s disease (HD), is the accumulation of misfolded, aggregation-prone proteins or peptides, essentially making them the veterinary horse of proteostatic disease. In some cases, such as in Alzheimer’s disease, the aggregates accumulate mainly extracellularly, forming plaques, while in others, such as Parkinson’s or Huntington’s disease, they form inside the affected neurons. Either way, these aggregates eventually lead to cell death and disruption of the neuronal tissue. In most of these pathologies, age is a leading risk-factor. This might be due to the age-related decline in the heat shock response and other elements of the neuronal proteostasis network [161]. N-terminal inhibitors of Hsp90 have been studied in the context of neurodegeneration resulting from a protein folding disorder. A high dose of 17-AAG was able to decrease intracellular neurofibrillary tangles (NFTs) in transgenic mice, while not affecting extracellular β-amyloid plaques [162]. Furthermore, Hsp90 inhibitors upregulated Hsp70, which in turn was able to prevent neuronal apoptosis [163]. Recently, Hsp70 was shown to be able to disassemble tau fibrils [164]. In *C. elegans,* however, it was reported that the disassembly of α-synuclein fibrils by the Hsp70 disaggregation machinery resulted in the generation of toxic amyloid-type protein species [165]. There are other promising small-molecule Hsp90-modulator candidates that have been tested in the context of neurodegenerative disease and heat shock response. One can refer to two excellent recent reviews for details [166,167]. A meta-analysis of the Alzheimer’s disease proteome revealed that the levels of Hsp90 family members is reduced in diseased brains, while most other chaperones and co-chaperones do not show change, with the notable exception of Hsp70, its nucleotide exchange factor BAG3, and neuronal small HSPs, which were found to be upregulated [168]. The upregulation of these components of the proteostasis machinery was suggested to be a last attempt by neurons to advert cell death.

For the vast majority of human diseases, aging has always been a significant risk factor. One thing that is common in most aging theories is the accumulation of molecular harm, including protein damage and dysfunction. Actually, loss of proteostasis is one out of nine hallmarks of aging [169]. Among the known modulators of lifespan through proteostasis, there are several Hsp90-dependent pathways, which may explain the pro-longevity effect of Hsp90 [66,170,171]. HSF1 has been associated with regulating the aging process for a long time [172], and has been shown to be necessary for the normal rate of aging and lifespan-extension in nematodes [173,174]. Likewise, FOXO transcription factor has already been mentioned multiple times. Besides its regulatory pathway being the first to be discovered to alter lifespan, it was also shown to be regulated by Hsp90 in *C. elegans* [66]. Many longevity genes are regulators of various stress responses [66,68,114]. The position of Hsp90 in this regard can almost be called unique, as it is not only part of a stress response pathway, but also contributes to the efficacy of other cytoprotective mechanisms, which eventually culminates in organismal survival through adequate responses to the challenges encountered in life.

Although most of these proteostatic diseases are predominantly intracellular, we have observed how cancer has extracellular Hsp90-dependent components, and the formation of toxic protein aggregates in neurodegenerative maladies also has consequences outside the cell. The examples described in the previous sections illuminated that these intra- and extracellular processes are often regulated by organism-level networks. The role and significance of Hsp90 in these pathways is not yet well understood, but future studies will bring us even closer to a more complete understanding.

## 8. Outlook and Concluding Remarks

Hsp90 has a diverse and context-dependent function in the maintenance of the proteome. It is essential to all three forms of intracellular proteostasis machineries. Other stress-response regulators, such as SIRT1 and FOXO, also depend on Hsp90 function, as well as the integrated stress response. As part of the complex chaperome assemblies of heat-shock proteins, co-chaperones and other co-factors, Hsp90 is a stabilizing force inside the cell. Outside of it, Hsp90 has multiple functions through binding to extracellular clients involved in ECM organization, or by binding to several receptors, leading to the activation of immune- or antioxidant-processes, or even wound healing. At the level of the organism, Hsp90 levels in one tissue can affect the expression, and thus the function of Hsp90 in distant tissues. As an evolutionary buffer that is capable of masking the effects of gene mutations, Hsp90 also has a phenotypic stabilizer function during development, while its capacity is tightly linked to phenotypic diversity necessary for continued population fitness. Its importance in various diseases, such as obesity, cancer, neurodegenerative disease and aging, is well demonstrated by the continuous development of small molecule Hsp90 modulators in the context of medical applications.

Several questions still remain concerning the role that Hsp90 plays in the maintenance of proteostasis. Little is known about how the different methods for Hsp90 secretion participate and interact with each other in the regulated processes. Likewise, further systematic studies are needed to explore the effects of eHsp90 on different target cells. In addition, while many studies were conducted using simple model systems, there is less information about how the relationships and uncovered Hsp90 inter-tissue signaling translate to higher organisms, or even humans. Research that identifies the tissue-specific Hsp90 interactomes in the organism and their changes in the response to various stresses, or the differences between intra- and extracellular interactome in different living systems, might provide further insights into these intricate processes.

## Figures and Tables

**Table 1:** Hsp90 interactors involved in proteostasis.

Hsp90 Interactors in Proteostasis				
Location	Process Affected	Name	Function	Reference
Intracellular	HSR	HSF1	Transcription factor	[76]
		Hsp70	Chaperone	[76]
		Hsp40	Chaperone	[76]
		p23	Co-chaperone	[77,78]
		Aha1	Co-chaperone	[77]
		Cdc37	Co-chaperone	[79]
		FKBP1	Co-chaperone	[80]
		FKBPL	Co-chaperone	[81]
		TTC4	Co-chaperone	[82]
		Hop	Co-chaperone	[77]
	UPR^ER^	PERK	Kinase, client	[4]
		IRE1	Kinase, client	[4,83]
	UPR^MT^	GCN2	Kinase, client	[69]
	Transcription regulation	SIRT1	Deacethylase, client	[68]
	Insulin/IGF1 signaling	DAF-16	Transcription factor	[66]
Extracellular	Wound healing/inflammation	CD91/LRP1	Receptor	[84]
	APC	LOX-1	Receptor	[85]
		SREC-1	Receptor	[85]
	Cell migration	Cdc37	Co-chaperone	[86]
	Activating Hsp90–MMP2 complex	Aha1	Co-chaperone	[87]
	MMP2 activation	p23	Co-chaperone	[88]
		Hop	Co-chaperone	[87,88]
		Hsp70	Chaperone	[88,89]
	ECM formation	Fibronectin	ECM glycoprotein, client	[90]
	Cell migration/metastasis Formation	MMP1	Endopeptidase, client	[91]
		MMP2	Endopeptidase, client	[92]
		MMP3	Endopeptidase, client	[93]
		MMP9	Endopeptidase, client	[94]

## Data Availability

Not applicable.
